# Long-Term Patency Rates of Portal Vein/Superior Mesenteric Vein Reconstruction after Pancreatic Resection for Pancreatic Tumors: Single-Center Experience

**DOI:** 10.3390/life14091175

**Published:** 2024-09-18

**Authors:** Miroslav Tomas, Peter Dubovan, Jana Pavlendova, Ramadan Aziri, Miroslav Jurik, Robert Duchon, Michal Bernadic, Nina Novotna, Jozef Dolnik, Daniel Pindak

**Affiliations:** 1 Department of Surgical Oncology, National Cancer Institute Bratislava, Klenova 1, 833 10 Bratislava, Slovakia; miroslav.tomas@nou.sk (M.T.); jana.pavlendova@nou.sk (J.P.); ramadan.aziri@nou.sk (R.A.); miroslav.jurik@nou.sk (M.J.); robert.duchon@nou.sk (R.D.); michal.bernadic@nou.sk (M.B.); nina.novotna@nou.sk (N.N.); jozef.dolnik@nou.sk (J.D.); daniel.pindak@nou.sk (D.P.); 2 Department of Surgical Oncology, Faculty of Medicine, Slovak Medical University, Klenova 1, 833 10 Bratislava, Slovakia

**Keywords:** pancreatic resection, venous resection, pancreatic cancer, reconstruction, thrombosis

## Abstract

To achieve an R0 resection margin in patients with locally advanced pancreatic ductal adenocarcinoma, high-volume pancreatic centers standardly incorporate portal vein or superior mesenteric vein resection. However, there is currently no consensus on the optimal reconstructive approach. Postoperative venous thrombosis or stenosis can significantly increase patient morbidity or mortality. The objective of this study was to report the long-term patency rate of portal/superior mesenteric vein reconstruction, as well as to identify potential predictors of postoperative venous thrombosis/stenosis. A single-center retrospective cohort analysis was conducted on patients undergoing pancreatic resection due to pancreatic tumor. The patency of the vascular reconstruction was assessed by routine surveillance using computed tomographic imaging at 3, 6, 9, and 12 months after surgery. A total of 297 pancreatic resections were performed with 53 patients undergoing concomitant venous resection. Among these, 26.4% (N = 14) had primary closure, 22.7% (N = 12) underwent an end-to-end anastomosis, and 50.9% (N = 27) received an interposition graft reconstruction. At the 1-year follow up, 90.2% (N = 37) of patients with venous reconstruction had a fully patent vein. The analysis did not reveal any statistically significant perioperative or postoperative factors associated with an increased risk of reconstruction thrombosis. While our study confirms a high long-term patency rate of 90.2% at 1 year, it underscores the necessity for a randomized controlled trial to determine the optimal method of venous reconstruction in pancreatic surgery.

## 1. Introduction

Pancreatic resection provides the only chance of cure for patients with pancreatic cancer. The proximity of the pancreatic head to the major vessels often results in tumor abutment, encasement or direct invasion with obstruction of the superior mesenteric vein (SMV) and portal vein (PV). Venous resection (VR) and reconstruction of the portal and superior mesenteric vein are occasionally necessary for the complete resection of pancreatic head tumors, and in high-volume centers, concomitant VR can be required in up to one-quarter of patients undergoing surgical resection, while offering survival rates comparable to those not requiring venous resection [1]. Consequently, the consensus statement from the International Study Group of Pancreatic Surgery (ISGPS) recommends primary operative exploration and resection in cases of reconstructable portomesenteric venous axis involvement [2]. Recent studies indicate that survival rates for patients undergoing VR during radical surgery for pancreatic head cancer are comparable to those undergoing conventional pancreaticoduodenectomy (PD). When a radical resection is achieved, VR in pancreatic cancer is a safe surgical procedure with equivalent long-term survival rates [3,4,5]. However, the recently published meta-analysis by Filho et al. reported a higher risk of short-term complications and mortality for PD combined with VR, along with a lower probability of survival compared to pancreatectomy without venous resection [6].

Even though achieving negative margins through VR may theoretically improve long-term survival, the potential higher risk of short-term complications and the lower probability of survival, with the results of PREOPANC trial and others favoring patients after neoadjuvant therapy, have shifted current therapeutic concepts from upfront resection towards neoadjuvant treatments in borderline resectable pancreatic cancer, with a continued search for the ideal therapeutic regimen [7,8,9]. Furthermore, with improved systemic treatment, we see a change in the treatment planning for patients with locally advanced pancreatic cancer, where patients once deemed unresectable may be able to undergo curative resection potentially in combination with vascular resection [9,10].

The current literature, however, lacks an evidence-based review that ponders the perioperative risks and the long-term survival rates associated with major vessel resection during PD for pancreatic cancer. Moreover, there is no consensus on the optimal method of vascular reconstruction. The techniques for vascular reconstruction in pancreatic surgery are generally classified into two main types: tangential resection with primary closure (venorrhaphy or patch venoplasty) and segmental resection with primary end-to-end anastomosis or graft interposition (Table 1) [11,12]. Recognizing the need for more evidenced-based data of venous reconstructions, the ISGPS proposed a classification of venous resections to standardize nomenclature for improved recommendations in the future (Table 1) [2]. Few studies have examined the long-term patency of the superior mesenteric-portal vein confluence following reconstruction.

## 2. Methods

**Figure 1:**
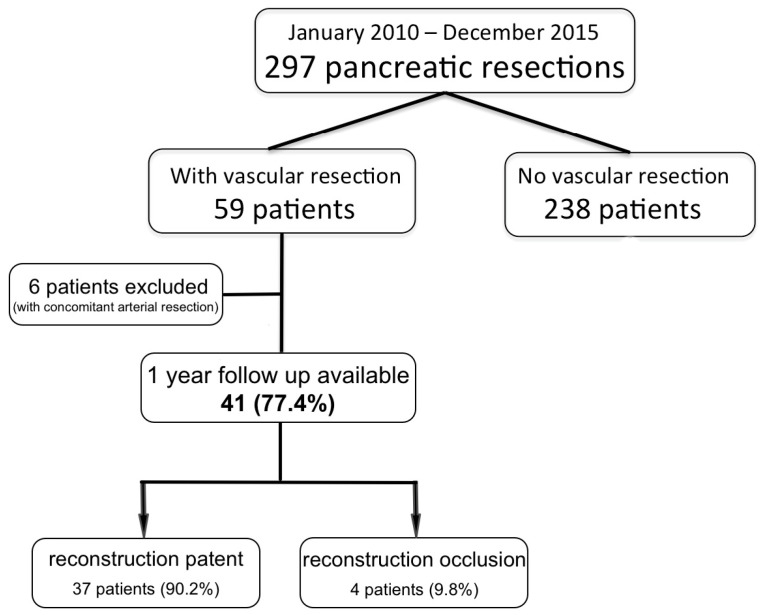
Study design and patient selection.

This retrospective cohort study evaluated the long-term patency of venous reconstruction following venous resection in patients with pancreatic tumors. The study encompassed patients who underwent surgery at the National Cancer Institute in Bratislava between 1 January 2010 and 31 December 2015. Patients were selected based on the requirement for the simultaneous vascular resection. During this period, 297 pancreatic resections were performed, with 59 patients undergoing simultaneous vascular resection. Of these, 6 patients who had simultaneous arterial and venous resection were excluded from the analysis. The remaining 53 patients were monitored with follow-up visits every three months. At 12 months after surgery, patients with available data were divided into two cohorts based on the patency of their venous reconstruction. At the 12-month follow-up, 37 patients exhibited patent venous reconstruction, forming the patent venous reconstruction cohort. Conversely, 4 surviving patients demonstrated non-patent venous reconstruction, constituting the non-patent cohort. The study design and patient selection are shown in Figure 1. Informed consent was not obtained for this study as all data were kept anonymous and personal health information was not revealed.

### 2.1. Surgical Technique and Characteristics

The predominant performed procedures were total pancreatectomy and the standard Whipple procedure. During the clamping of the mesenterico-portal veins, conventional heparin was administered at a dose of 100 units per kilogram. Continuous anticoagulation therapy was subsequently provided only to patients who underwent prosthetic reconstruction. For the patients undergoing total pancreatectomy that required interruption of the left gastric vein, a standard subtotal gastrectomy was added to ensure good venous drainage of the remaining stomach.

### 2.2. Statistical Analysis

The statistical evaluation of differences was conducted based on the long-term patency assessed 12 months after surgery for the entire cohort. All statistical hypotheses were tested at a significance level of α = 0.05. The statistical relationships were evaluated by the *t*-test, Mann–Whitney U test, Fisher exact test, Fisher–Freeman–Halton test, and Chi-square test. Differences in Clavien–Dindo classification were assessed using the non-parametric Mann–Whitney U test and Kolmogorov–Smirnov test. Statistical analyses were performed using the software Statsdirect version 3.0.177.

## 3. Results

A total of 53 patients underwent pancreaticoduodenectomy, distal pancreatectomy, or total pancreatectomy with vein resection for pancreatic head and periampullary neoplasms. Among these, 14 patients (26.4%) underwent venous resection with primary closure, 12 patients (22.7%) had an end-to-end anastomosis, and in 27 cases (50.9%), an interposition graft was used. Of these patients, 41 patients were alive with available data on venous reconstruction patency 12 months after surgery. At the one-year follow-up, 37 patients (90.2%) had patent vascular reconstructions, while 4 cases (9.8%) experienced occlusion of the vascular reconstruction.

**Gender.** In the cohort with patent venous reconstruction, there were 20 males (54.1%) and 17 females (45.9%). The non-patent venous reconstruction cohort comprised two males (50%) and two females (50%). The difference between these two cohorts was not statistically significant (*p* > 0.9999).

**ASA classification grade.** The preoperative ASA classification of the patient cohorts showed no significant difference (*p* = 0.30) with an even distribution within the ASA scale classification 1–3. No patients were ranked as ASA 4 [13].

**Nakao classification grade.** Preoperative assessment of venous infiltration by the primary tumor was based on the CT scans, categorizing patients into four groups using the Nakao classification [14]. In the cohort with patent reconstruction, the most common type of infiltration was type B in 22 cases (59.5%), followed by type C in 11 cases (29.7%) and type D in 4 cases (10.8%). In the non-patent venous reconstruction cohort, all four cases (100%) had type C infiltration. There was no statistically significant difference between the cohorts regarding the type of infiltration based on Nakao classification (*p* = 0.51).

**Type of surgery.** In the cohort of patients with a patent venous reconstruction, the types of performed surgery were total pancreatectomy in 25 cases (67.6%), Whipple procedure in 11 cases (29.7%), and distal pancreatosplenectomy in 1 case (2.7%).

In the non-patent venous reconstruction cohort, the performed surgeries were Whipple procedure in two cases (50%) and total pancreatectomy in two cases (50%). Statistical analysis did not show a significant difference between the cohorts regarding the type of surgery (*p* = 0.59). All group characteristics are summarized in Table 2, and detailed surgical information is provided in Table 3.

**Figure 2:**
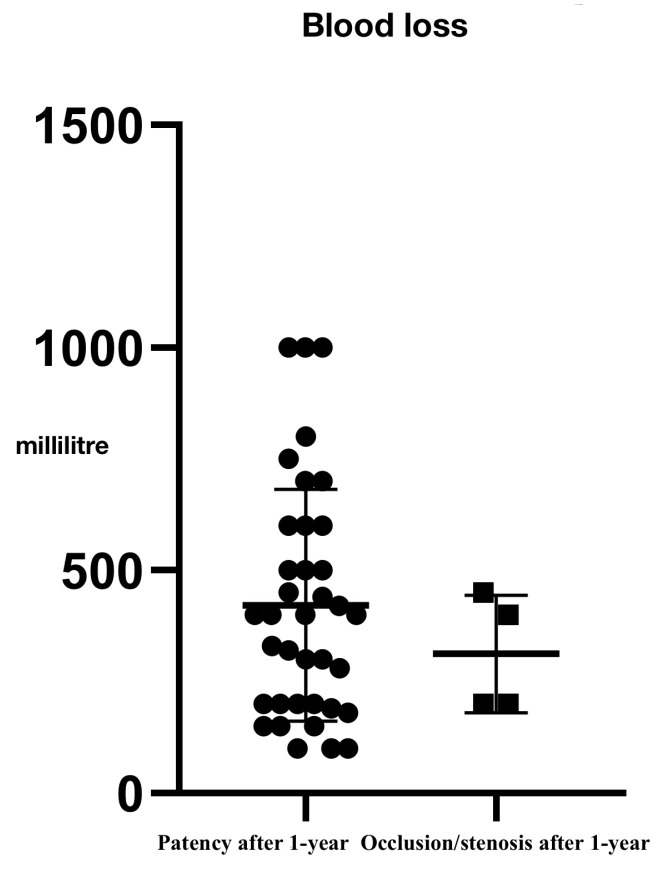
Blood loss.

**Blood loss.** In the cohort of patients with patent venous reconstruction, the blood loss ranged from 100 to 1000 mL, with a median of 400 mL and an average of 421.9 mL. In contrast, in the cohort with non-patent reconstruction, the blood loss ranged from 200 to 450 mL, with a median of 300 mL and an average of 312.5 mL. The Mann–Whitney U test showed no statistically significant difference between these groups (*p* = 0.6345), as illustrated in Figure 2.

**Figure 3:**
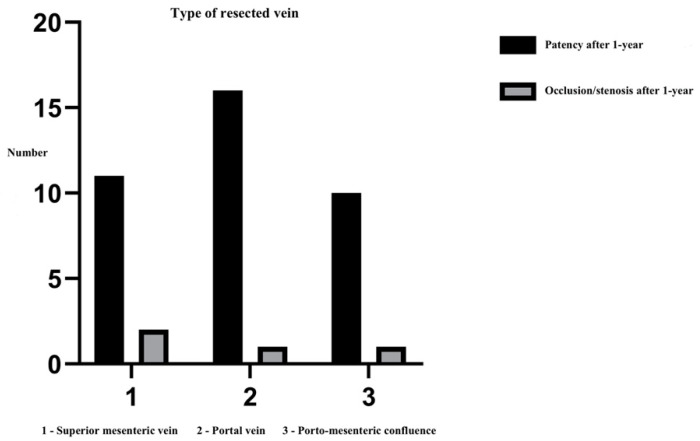
Type of resected vein.

**Type of resected vein.** The resection of the superior mesenteric vein, portal vein, or mesenterico-portal vein was conducted based on the localization of the infiltration. Among patients with a patent reconstruction, resection of the portal vein was performed in 16 cases (43.3%), followed by resection of the superior mesenteric vein in 11 cases (29.7%) and resection of the venous confluence in 10 cases (27%). In the cohort with an occluded reconstruction, resection of the superior mesenteric vein was performed in two cases (50%), segmental resection of the portal vein in one case (25%), and resection of the venous confluence in one case (25%). Notably, statistical analysis of these data did not reveal significant relevance (*p* = 0.710, Figure 3).

**Vascular clamp duration.** In the cohort with patent reconstruction, vascular clamping durations ranged from 7 to 40 min with a median of 20 min and an average of 22.3 min. In the cohort with occluded reconstruction, clamping durations ranged from 15 to 45 min, with the median and average of 30 min each. Statistical analysis revealed no significant difference between the cohorts (*p* = 0.2434).

**Reconstruction method.** Within the cohort of patent reconstructions, a prosthetic graft interposition bypass was used in 17 cases (45.9%), while primary suture or end-to-end anastomosis was utilized in 20 cases (54.1%). In the occluded reconstruction cohort, graft interposition was used in three cases (75%) and primary anastomosis in one case (25%). Statistical analysis demonstrated no significant difference between the cohorts (*p* = 0.3433).

**Postoperative morbidity.** Postoperative morbidity was classified according to the Clavien–Dindo classification (Table 4) [15]. The Chi-square test was conducted to assess the statistical significance of postoperative morbidity in groups with patent and occluded venous reconstruction. The observed difference between cohorts did not reach statistical significance (*p* = 0.0846).

**Figure 4:**
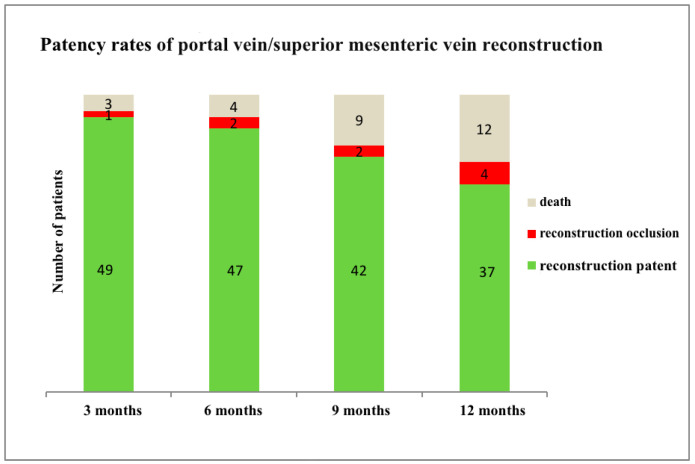
Long-term patency rates of portal vein/superior mesenteric vein reconstruction.

**One-year patency of reconstructions.** The patency of venous reconstruction was systematically assessed at 3-month intervals following surgery with a comprehensive evaluation and statistical analysis conducted at the one year mark (Figure 4). Initially, 53 patients underwent pancreatic resection with concomitant venous resection. Ultimately, data from 41 patients were available for analysis at the 12-month milestone. At the one-year evaluation, occluded vascular reconstruction was observed in 4 patients (9.8%), while 90.2% (37 patients) demonstrated continued patency of venous reconstruction.

## 4. Discussion

Venous resection and reconstruction of the portal vein or superior mesenteric vein are occasionally required for the complete resection of pancreatic tumors. Over the past few decades, clinical outcomes following pancreatic surgery have considerably improved, with a reduction in postoperative morbidity and mortality. Previous studies indicated no significant differences in terms of postoperative morbidity and long-term survival after pancreatectomy with PV-SMV resection [3,4]. However, a meta-analysis by Giovinazzo et al. (2016) reported the significantly inferior 1-, 3-, and 5-year survival rates of patients undergoing PD with VR. This analysis, which encompassed 27 studies and 9005 patients (including 1587 undergoing pancreaticoduodenectomy with venous resection), also identified a heightened risk of postoperative complications, reoperations, and postoperative mortality [16]. A more recent meta-analysis by Zwart et al. (2022) echoed these findings, revealing significantly lower survival rates at 1, 3, and 5 years among patients undergoing pancreatic resection with venous resection. While no statistically significant differences were observed in postoperative pancreatic fistula, delayed gastric emptying, or reoperation rates, the analysis did indicate a higher incidence of postpancreatectomy hemorrhage and 30-day mortality in the group undergoing venous resection. Notably, there was no difference in 90-day mortality between groups. Despite these findings, concomitant venous resection continues to be regarded as a safe and feasible option in patients undergoing pancreatic resection with regard to current clinical guidelines predominantly after neoadjuvant treatment [9,17].

Currently, there exists no consensus regarding the optimal reconstructive approach for VR during pancreatic surgery. Nevertheless, it is imperative to note that postoperative venous thrombosis or stenosis may contribute to elevated morbidity and mortality rates among patients. Despite this significance, only a few studies have examined the long-term patency of the superior mesenteric–portal vein confluence following reconstruction [18,19]. Several vascular reconstruction techniques have been extensively documented in the literature using a variety of materials, ranging from synthetic to biological. Notably, the study by Gao et al. revealed superior long-term venous patency and lower infection rates associated with the utilization of primary or native venous grafts (88–93%) compared to those using synthetic composition grafts (68–74%) [20]. Among the synthetic grafts, polytetrafluoroethylene (PTFE) stands as the most widely used [21], yet it presents several drawbacks, such as a high risk of thrombosis, the requirement of long-term anticoagulation, and the risk of infection. In contrast, biological grafts such as cryopreserved venous allografts [22], autologous falciform ligaments [23], or bovine grafts [24] offer potential advantages, such as the reduced risk of infection and decreased need for anticoagulation. Nevertheless, they often come at a higher cost and are subject to limited availability. A case report published by Akimaru et al. described the successful utilization of peritoneum as an autologous vascular graft for the reconstruction of other major vessels in hepato-biliary surgery [25]. The largest existing series to date, comprising 52 patients, was reported by the French group led by Dokmak. In this study, the application technique utilizing parietal peritoneum as a graft for the reconstruction of the mesentericoportal vein axis, particularly following pancreatic resections for malignant disease, was presented for the first time. The ‘Safi Dokmak Vascular Graft’ presented a good overall patency rate of 96% at 11 months of mean follow-up [26]. Autologous peritoneal flaps utilized as venous substitutes present several advantages, such as a cheaper cost, the absence of thrombogenic risk, great versatility and easy adaptation to the vascular defect size, minimal additional operative time requirements, and immediate availability, even in the emergency setting [27]. The objective of the present retrospective study was to assess perioperative outcomes and long-term reconstruction patency in patients who received vascular reconstruction for PV-SMV reconstruction subsequent to pancreatic resection with VR. A study using only autologous interposition grafts by Tseng et al. referred to a patency rate of reconstruction of 92% at 1 year after resection [12]. A study by Fujii et al. retrospectively evaluated 197 patients who underwent VR simultaneously with pancreatectomy to determine postoperative patency. In their series, 18 (9%) patients developed severe anastomotic stenosis (≥70% occlusion). Univariate analysis revealed prolonged operative time (520 min) and length of vascular resections (>31 mm) to be associated with the development of severe stenosis. Data from this series provide the incidence and timing of vein thrombosis following PD with VR, yet offer limited clarity on the underlying factors contributing to venous thrombosis. There was no difference in venous clamping time length among patients with vein thrombosis [28]. These findings align with our study results, wherein we observed shorter vascular clamp times in the cohort of patent venous reconstruction (22.3 ± 9.96 min vs. 30.0 ± 12.9 min), although this disparity did not attain statistical significance (*p* = 0.2434). In our study, the statistical analysis was performed based on the evaluation of the one-year patency of the reconstructions and the further division of patients into two cohorts. The analysis of the patency of the venous reconstructions was carried out based on CT scans within the scope of the oncological dispensary care of patients 3, 6, 9, and 12 months after surgery. The evaluation of CT scans 3 months after surgery was available for 50 patients, since 3 patients passed away before the initial follow-up assessment. There was no case of 30-day mortality associated with occlusion of the venous reconstruction. The cause of 90-day mortality in two patients was attributed to septic shock with multi-organ failure, while in the third case, the death of the patient resulted from severe necrotic pancreatitis subsequent to the Whipple procedure with venous resection. The nine remaining patients for whom there were unavailable data regarding venous reconstruction patency after 12 months died due to cancer progression in the period leading to the 12 month mark. There were no instances of mortality attributed to acute obstruction or thrombosis of the reconstructed vein. Among 53 patients who underwent pancreatectomy with VR, 23 (44%) had tumors involving the portal vein, 16 (30%) had tumors involving the SMV, and 14 (26%) had tumors involving the mesenterico-portal confluence. Reconstruction by primary anastomosis and venorraphy was performed in 26 cases, while a prosthetic interposition graft was used in 27 cases. No peritoneal patches were employed. Among the analyzed 50 patients, venous reconstructions remained patent in 98% of living patients at 3 months, 95.9% at 6 months, 95.5% at 9 months, and 90.2% at 12 months (Table 5). 

These results regarding thrombosis and patency rates are consistent with recent high-volume specialized single-center reports, which have demonstrated the feasibility, safety, and high one-year patency rate of the combination of pancreatic resection with venous resection [12,29].

The outcomes of this study should be interpreted with caution due to several limitations, including the relatively small sample size of our cohorts, potential biases, and the low incidence of thrombotic events. Among 53 patients in our series who underwent pancreaticoduodenectomy with VR, we observed four thrombotic/occlusion events resulting in a thrombosis rate of 9.8%. One study reported a portal vein thrombosis rate of 10%, with the highest incidence in those undergoing an end-to-end anastomosis [30]. Another study, which also predominantly utilized primary closure (45%), found no significant difference in the incidence of portal vein thrombosis between the three groups (primary end-to-end repair—autologous vein graft—PTFE graft) and reported an overall thrombosis rate of 17% [31]. Song et al. conducted a meta-analysis revealing that primary end-to-end anastomosis was the most frequent procedure, performed in 570 patients (68.1%), followed by synthetic vein grafts in 131 patients (16.6%), autologous vein grafts in 110 patients (13.1%), allograft vein grafts in 14 patients (1.7%), and other materials in 4 patients (0.5%). The authors reported no significant differences in perioperative morbidity and mortality or thrombosis between groups with and without grafts. However, the group with vein grafts was associated with a significantly increased venous thrombosis rate at six months or more (OR = 2.75; CI 1.32–5.73). The subgroup analysis revealed a significantly increased rate of venous thrombosis in the group with autologous vein grafts compared to those with prosthetic vein grafts [32]. Ravikumar et al. reported the portal vein thrombosis rate of 4.4%. In the same study, interposition graft reconstruction was associated with an increased risk of developing portal vein thrombosis, potentially due to the greater technical complexity of this reconstruction method [33]. Kang et al. reported long-term patency rates, with a stenosis/occlusion rate of 19,6% following pancreatoduodenectomy with reconstruction and a 5-year patency rate of 69.9%. The most frequent causes of stenosis/occlusion were local recurrence followed by postoperative intra-abdominal changes and PV thrombosis. The 3-year patency rate was highest in patients with cancer of the ampulla of Vateri and lowest in patients with pancreatic cancer (91.9% versus 55.5% respectively; *p* < 0.001). Multivariable analysis identified several risk factors for thrombosis including primary tumor location, chemoradiotherapy, and PV resection. Portal vein stenosis or occlusion without disease recurrence was observed in 17.3% of the patients. Independent risk factors for PV stenosis or occlusion included PV resection and grade B or C postoperative pancreatic fistula [34]. In our study, we found out that the length of clamping (*p* = 0.24), amount of blood loss (*p* = 0.63), tumor localization (*p* = 0.71), type of reconstruction (*p* = 0.34), and severity of postoperative complications (*p* = 0.07) did not significantly affect the long-term patency rates of the reconstructions provided that standard postoperative antithrombotic and anticoagulation guidelines were followed. A recently published systematic review by Labori et al. [35] highlighted the lack of consensus on the definition and reporting of long-term graft patency following pancreatectomy with SMV-PV resection and reconstruction. The review reported early and overall graft thrombosis incidence of 7.5% and 22.2% for synthetic grafts, 5.6% and 11.7% for autologous vein grafts, 6.7% and 8.9% for autologous parietal peritoneum/falciform ligaments, and 2.5% and 6.2% for allografts. The authors concluded that autologous, allogenic, or synthetic grafts for SMV-PV reconstruction are safe and feasible in selected patient groups, although a higher rate of graft thrombosis was observed in synthetic grafts.

Limitations of this study include the small sample size of patients with occlusion of the vascular reconstruction. This small sample size of patients hindered a definitive statistical evaluation of the differences, resulting in weak statistical test power.

## 5. Conclusions

Our study confirmed a high long-term patency rate of 90.2% at 1 year after surgery, underscoring the reliability of our surgical technique within our patient cohort. We have observed that occlusion of the reconstruction exhibited no statistically significant correlation to the type of reconstruction, localization of vein resection, duration of intraoperative clamping, blood loss, and intraoperative hemosubstitution in the perioperative period (*p* = 0.3024) and postoperative period (*p* > 0.9999), as well as perioperative morbidity. However, further randomized, controlled studies are necessary to assess and obtain any evidence of differences existing among the different techniques and to eventually support its superiority over other more diffusely used vascular reconstruction options.

## Figures and Tables

**Table 1:** ISGPS classification of venous resections.

Type	Classification
I	Partial venous excision with direct closure by suture closure (venorraphy)
II	Partial venous excision using a patch (venoplasty)
III	Segmential resection with primary veno-venous anastomosis end-to-end
IV	Segmental resection with interposed venous conduit (graft) and at least two anastomoses

**Table 2:** Cohort’s characteristics.

	Cohort 1 Patency after 1 Year	Cohort 2 Occlusion/Stenosis after 1 Year	*p* Value
Number (n/%)	37/90.2	4/9.8	
Gender (male/female %)	54.1/45.9	50.0/50.0	>0.9999 ^1^
ASA classification grade 1/2/3/4 (n)	2/23/12/0	0/4/0/0	0.3056 ^1^
Nakao classification grade B/C/D (%)	59.5/29.7/10.8	0/100.0/0	0.5166 ^1^
Total Pancreatectomy/Pancreatoduodenectomy/Distal Pancreatosplenectomy (n/%)	25 (67.6)/11 (29.7)/ 1 (2.7)	2 (50.0)/2 (50.0)/0	0.5956 ^1^

^1^ Mann–Whitney U test, n number.

**Table 3:** Surgical data.

	Cohort 1 Patency after 1 Year	Cohort 2 Occlusion/Stenosis after 1 Year	*p* Value
Type of operation n (%)			0.5956 ^1^
PD	11 (29.7)	2 (50.0)	
TP	25 (67.6)	2 (50.0)	
DP	1 (2.7)	0	
Surgery duration (min, mean ± SD)	395.5 ± 78.1	416 ± 179.3	0.6941 ^1^
Blood loss (mL, mean ± SD)	421.9 ± 260.1	312.5 ± 131.5	0.6345 ^1^
Type of resected vein			0.710 ^2^
PV	16 (43.3)	1 (25.0)	
SMV	11 (29.7)	2 (50.0)	
PV-SMC confluence	10 (27.0)	1 (25.0)	
			
Type of reconstruction n (%)			0.3433 ^3^
Interposition graft (PTFE)	17 (45.9)	3 (75.0)	
No interposition graft	20 (54.1)	1 (25.0)	
			
Vascular clamp time (min, mean ± SD)	22.3 ± 9.96	30.0 ± 12.9	0.2434 ^1^
			
Multivisceral resection	6 (16.2)	0	>0.9999 ^3^
			
Perioperative complications			
POPF			>0.9999 ^3^
Grade A	3	0	
Grade B	0	0	
Grade C	0	0	
PPH			0.0952 ^1^
Grade A	1	1	
Grade B	2	1	
Grade C	5	0	
Postoperative infectious complications			
Intra-abdominal abscess	2	0	>0.9999 ^1^
SIRS/sepsis	13	1	>0.9999 ^3^
			
Surgical reoperation n (%)	6 (12.5)	1 (20.0)	0.3478 ^1^
			
30-day mortality n (%)	2 (5.4)	0	0.99 ^3^

SD: standard deviation, n: number, PD: pancreatoduodenectomy, TP: total pancreatectomy, DP: distal pancreatectomy, PV: portal vein, SMV: superior mesenteric vein, PTFE: polytetrafluoroethylene, POPF: postoperative pancreatic fistula, PPH: postpancreatectomy hemorrhage, SIRS: systemic inflammatory response syndrome. ^1^ Mann–Whitney U test, ^2^ G-square test, ^3^ Fisher’s exact test.

**Table 4:** Morbidity according to Clavien–Dindo classification showed no significant difference between groups.

Clavien–Dindo	Cohort 1 Patency after 1 Year n = 37 (n/%)	Cohort 2 Occlusion/Stenosis after 1 Year n = 4 (n/%)	*p* Value
1	13/35.1	2/50.0	
2	16/51.4	0	
3a	2/5.4	1/25.0	
3b	3/8.1	1/25.0	
4a	3	0	0.0846 ^1^
4b	0	0	
5	0	0	

^1^ Chi-square test.

**Table 5:** Patency over time.

Time Point	Baseline	3 mo	6 mo	9 mo	12 mo
Rate of thrombosis (%)	0%	2%	4.1%	4.5%	9.8%
Rate of patency (%)	100%	98%	95.9%	95.9%	90.2%
Total patients with thrombosis (n)	0	1	2	2	5
Total alive patients with thrombosis (n)	0	1	2	1	4
Total alive patients PD + VR (n)	53	50	49	44	41
Percentage of original patients still alive (%)	100%	94.3%	92.5%	83%	77.4%

## Data Availability

The original contributions presented in the study are included in the article; further inquiries can be directed to the corresponding author.
